# Supplementary material for: Foundation model enables interpretable open and error-tolerant searching for mass spectrometry-based proteomics
Source: Bioinformatics. 2026 Jul 7;42(Suppl 1):btag297. doi: 10.1093/bioinformatics/btag297 (PMC13340164; doi:10.1093/bioinformatics/btag297)
Supplement: btag297_Supplementary_Data [file btag297_supplementary_data.pdf]

Supplementary Material

Preprocessing and encoding of spectra and peptides

The intensities are normalized such that the intensities $(I_1, ..., I_l, ..., I_N)$ are a unity-length vector (i.e. scaled by $1/\sqrt{\sum I_l^2}$). For each peaks list, up to 500 peaks are kept. Otherwise, if the list contains more than the maximum number of peaks, we keep peaks according to the top-500 highest intensities or append tuples of (0.0,0.0) if the peaks list contained less peaks.

Both spectra and peptides are fed to Transformer architectures, hence we adapt the original positional encoding (also called wavelet encoding) and extend it to suit the context of spectra and peptides.

For the positional encoding of the peaks list of the peaks list $(mz_1, ..., mz_l, ..., mz_{500})$, each mz_l is multiplied by the radiant rate r_i of the i-th dimension of the encoding vector s: $s_{li} = mz_l \cdot r_i$, where $r_i := 10000.0^{-i/d}$. Furthermore, $S'_{li} = sin(s_{li})$ if i is an even integer and $S'_i = cos(s_i)$ if i is odd. For the final spectrum encoding S, each positional encoded mz_l is scaled by its respective intensity: $S_i = I_l \cdot S'_{li}$. Hence S is a tensor of size (number of peaks, d) = (500,64).

For each peptide sequence, we enumerate each amino acid (starting from zero, up to peptide length n - 1) these integer positions p_n are then multiplied by a radiant rate r_i (same definition as above) of the i-th dimension of the peptide positional encoding vector p: $p_{li} = p_l \cdot r_i$. Up to n=42 amino acids are allowed, if the peptide is shorter it is padded with zeros. Hence, the peptide positional encoding vector has a size of (42,64). Furthermore, the amino acids are replaced by unique indices (starting from 0 up to 25, with zero being a padding character) and function as the index into a lookup table of size (alphabet size, d) = (26, 64), where the parameters of the lookup table are trainable and trained together with the model. For the final peptide sequence encoding P, each positional encoded p_i is multiplied by its amino acids embedding (from the lookup table) : $P_{li} = p_{li} \cdot aa_i$. Hence P is a tensor of size (maximum peptide length, d) = (42,64).

Training data

The Transformer models of yHydra were trained on 19,991,263 PSMs (retrieved as USIs) from the following 67 repositories: PXD000702, PXD001072, PXD001344, PXD001351, PXD002054, PXD002147, PXD003094, PXD003261, PXD003364, PXD003556, PXD003718, PXD003779, PXD003916, PXD003976, PXD004398, PXD004825, PXD005009, PXD005117, PXD005196, PXD005306, PXD005341, PXD005654, PXD005744, PXD006033, PXD006084, PXD006316, PXD006375, PXD006389, PXD006645, PXD006823, PXD006836, PXD008592, PXD008602, PXD008622, PXD008647, PXD008667, PXD008895, PXD009387, PXD009665, PXD009698, PXD009713, PXD010000, PXD010641, PXD010827, PXD011042, PXD011583, PXD011712, PXD011714, PXD011984, PXD012827, PXD013274, PXD013304, PXD013684, PXD013711, PXD013712, PXD013724, PXD013890, PXD013897, PXD015153, PXD015296, PXD015698, PXD016833, PXD016846, PXD017308, PXD018714, PXD019095, PXD019134. We specifically selected repositories of non-model organisms (excluding the top-10 most commonly studied organisms in terms of counts of repositories) to reduce the bias towards certain proteome-specific sequence patterns. Furthermore, we only included data acquiered on Q Exactive.

Pairwise contrastive loss of yHydra

The loss of yHydra is inspired by the recent approach of CLIP [Radford et al., 2021] and is based on the idea to directly calculate a contrastive loss based on the pairwise distances within each mini-batch (table 2 and Fig.1A). We found this approach has major advantages over previous types of contrastive-losses or triplet-losses while virtually having none of their shortcomings. Most importantly the pairwise contrastive loss does not not require the creation of artificial negatives as they naturally occur due to the mixed pairs of distances (i.e. off-diagonal elements in Fig.1A). This is not only more elegant than previous contrastive loss formulations but also makes the network learn at anytime, whereas for older types of contrastive losses typically hard-negative mining was essential to get decent training results. Furthermore, we extended this idea by adding label smoothing which should allow the model to also learn from the specific but small distances that mixed negatives naturally have. Label smoothing allows the model to also learn from this regime (i.e. off-diagonal elements in Fig.1A).

IN	:	spectrum embedding $T_{SPEC}(SPEC_{ENC})$<br>peptide embedding $T_{SEQ}(SEQ_{ENC})$
E_{SPEC}	=	$L2Norm(T_{SPEC}(SPEC_{ENC}))$
E_{SEQ}	=	$L2Norm(T_{SEQ}(SEQ_{ENC}))$
D	=	$L2Dist_{pairwise}(E_{SPEC}, E_{SEQ}.T)$
$logits$	=	$-tf.log(D + eps) \cdot tf.exp(T)$
$targets$	=	$tf.range(N)$
L_{SPEC}	=	tf.sce(targets,logits,smoothing,axis=0)
L_{SEQ}	=	tf.sce(targets,logits,smoothing,axis=1)
$loss$	=	$(L_{SPEC} + L_{SEQ})/2$
OUT	:	$loss$

Table 2. Tensorflow-/Numpy-like pseudocode of the calculation of the pairwise loss between spectra and peptides. The Transformer models T_{SPEC} and T_{SEQ} yield embeddings E_{SPEC} and E_{SEQ} each of size (batch-size=64, embeddings-size=64). The spectrum encoding $SPEC_{ENC}$ and peptide sequence SEQ_{ENC} are described in the main text. Parameters are set to eps=0.001, smoothing=0.1 and T=3.0 and tf.sce is the sparse cross entropy between targets and logits according to the the distance matrix D (illustrated in Fig. 1A).

GPU-accelerated peak matching and PSM scoring

The core algorithms of yHydra are GPU-accelerated (i.e. neural networks and k-NN search by faiss [Johnson et al., 2019]). To further speed up the runtime of yHydra we developed a GPU-accelerated peak-matching and PSM scoring (table 3). The idea is to simultaneously score a batch of 64 spectra against their respective k-candidates, i.e. k=50, which means for 3,200 PSMs peaks are matched and scored in parallel.

			tensor size
IN	:	queries $q = mz_{theor}$	[b,k,l]
		keys $k = mz_{acq.}$	[b,n]
		values $v = I_{acq.}$	[b,n]
M	=	$L2Dist_{pairwise}(q, k.T)$	[b,k,l,n]
M	=	tf.where($M <$ tolerance, 1/(M), 0.0)	[b,k,l,n]
M	=	tf.reduce_maximum(M,axis=2)	[b,k,n]
M	=	tf.where($M > 0.0, 1.0, 0.0$)	[b,k,n]
S	=	$M \cdot v$	[b,k]
S	=	tf.reduce_maximum(S,axis=-1)	[b]
I	=	tf.argmax(S,axis=-1)	[b]
OUT	:	indices (I) and scores (S) of the best matching peptide for each spectra in the mini-batch of size b	

Table 3. Tensorflow-/Numpy-like pseudocode of peak matching and scoring for PSMs. The inputs are a mini-batch of b spectra, with n peaks considering their mz-values $mz_{acq.}$ and intensities $I_{acq.}$. Furthermore, list of k candidate peptide (result of the k-NN search) is considered as theoretical ions with up to l mz-locations mz_{theor}, see Methods for details on parameters.

Multiplexed k-NN search of mass buckets for closed, narrow and open searches

Our multiplexed k-NN search allows us to search all spectra embeddings against all peptide embeddings within the same search call while at the same time only spectra against theoretically possible peptides (e.g. determined by certain combinations of precursor mass and respective peptide masses) are searched. Therefore we divide the peptides in the database into buckets according to their theoretical mass. Hence, for a closed search we could have a thousand of small buckets (of +/-1 Da width) and, in contrast, for the open search we have a few but wide buckets (e.g. +/-500 Da width). Each bucket gets a unique vector assigned, which is appended to the peptide embeddings in that bucket (i.e. similar to an 'address' vector). Subsequently, the query embeddings, which are supposed to be searched against a specific bucket also gets the respective address vector appended. Effectively, the L2-norm between the embeddings is dictated by their common 'address'-vector because only those with a common address-vector have meaningful intra-buckets L2-distances but comparatively high inter-bucket L2-distances. Ultimately, this allows us to achieve multiple mass-compliant search calls while really only performing a single search.

Discussion of false discovery rate (FDR) for yHydra's beam search

The assumption of the target-decoy approach is that for a given set of reference protein sequences a false discovery rate can be estimated by generating a complementary set of decoy sequences (randomized or reversed sequences) of similar size. Any hit against the decoy database is considered a false positive. However, the target-decoy approach has its limitations when the reference space becomes very large Renard et al. [2012], or even unlimited, as in case of de novo sequencing, for which there is no direct way to generate decoys Sanders et al. [2025].Ultimately, our approach sits in-between both scenarios: i) a classical database search, constrained to the search space of target and decoy sequences, and ii) de novo sequencing, which is unconstrained and thus adequate FDR-estimation remains limited. However, because our approach starts from the results of an initial open search against target and decoy sequences we are able to track how targets and decoys behave throughout our gradient-based decoding. Specifically, we forward targets and decoys, through our gradient-based error-tolerant search and thus show that the decoded peptides that originated from target sequences further improve their scoring, meaning the final decoded peptide better matches the underlying spectra. In contrast, the gradient-based decoded peptides, initialized from decoys, rarely improve their scores, and, in fact, decoys remain pinned, close to their original poor scores (Fig. 4B and Fig. 4F).

Regarding validation, there are at least two ways to validate newly suggested peptide sequences (that differ from previously known reference sequences). First, through in silico validation, that is predicting the fragment intensity for a suggested peptide and subsequently comparing the acquired fragment intensities against the fragment intensity predictions. Such in silico validation could be achieved by using machine learning-based methods like Prosit or ms2pip that are able to predict fragment intensities. Second, a validation can be achieved by synthesizing the newly suggested peptide species from ordering these peptides from commercial vendors.

Considerations of post-translational modifications (PTMs) in yHydra

We identify four different stages in which PTMs are crucial for yHydra:

During model training, the peptide sequence transformer receives unmodified peptide sequences. Thus yHydra needed to robustly learn to jointly embed spectra (from a potentially modified and/or unmodified peptide) to a common unmodified peptide sequence embedding. In fact we tested this and can show that yHydra embeds both unmodified and modified peptides in an unbiased manner (Fig. 1D). This is crucial for the open search.

During open searching, regardless of the PTM status, the Euclidean distance in the joint embedding space during searching should point to the true unmodified peptide sequence (minimal Euclidean distance between spectrum embedding and the sequence embedding). This is exactly why the delta mass becomes meaningful as the goal of the open search is to provide the best-matching unmodified peptide sequence and delta masses in an unbiased manner.

For the final scoring of our implementation of the yHydra closed searching it is possible for users to state a list of PTMs and corresponding delta masses (per each defined PTM), such as +79 Da for phosphorylations on amino acids such as S, T, and Y and a count for maximum allowed occurrences of that PTM in the sequence. The final scoring would consider additional, modified peptide species subjected to the closed search in yHydra. However, for an exploratory open search, i.e. the user does not specify any PTMs, the distribution of delta masses – as shown in our original manuscript provides an unbiased manner of exploring the space of potential PTMs contained in the dataset. Delta masses, per PSM, are defined as precursor mass minus the matching reference peptide mass.

During our beam search for the gradient-based decoding, we currently do not consider PTMs. We acknowledge that we could further improve our approach by including additional tokens to the peptide sequence embedder to reflect amino acids with PTMs explicitly during model training. Subsequently, this could be included in the beam search, e.g. to estimate site-specific probabilities of phosphorylations.

Code availability

An open source implementation with command-line instructions is publicly available (under MIT license) at `https://gitlab.com/dacs-hpi/yHydra`. A separate open source repository for training yHydra is available at `https://gitlab.com/dacs-hpi/yHydra_train`.

Data availability

Proteomic data were downloaded from public repositories PXD000702, PXD001072, PXD001344, PXD001351, PXD002054, PXD002147, PXD003094, PXD003261, PXD003364, PXD003556, PXD003718, PXD003779, PXD003916, PXD003976, PXD004398, PXD004825, PXD005009, PXD005117, PXD005196, PXD005306, PXD005341, PXD005654, PXD005744, PXD006033, PXD006084, PXD006316, PXD006375, PXD006389, PXD006645, PXD006823, PXD006836, PXD008592, PXD008602, PXD008622, PXD008647, PXD008667, PXD008895, PXD009387, PXD009665, PXD009698, PXD009713, PXD010000, PXD010641, PXD010827, PXD011042, PXD011583, PXD011712, PXD011714, PXD011984, PXD012827, PXD013274, PXD013304, PXD013684, PXD013711, PXD013712, PXD013724, PXD013890, PXD013897, PXD015153, PXD015296, PXD015698, PXD016833, PXD016846, PXD017308, PXD018714, PXD019095, PXD019134 to train yHydra. Furthermore, public data from PXD007963 was downloaded to evaluate yHydra. For the monoclonal antibody benchmark, data was downloaded from MSV000079801 via MassiveKB. The Chimpanzee plasma data is available as a Zenodo dataset Altenburg et al. [2024].
